# Chronic Diseases and Associated Risk Factors Among Adults in Puerto Rico After Hurricane Maria

**DOI:** 10.1001/jamanetworkopen.2021.39986

**Published:** 2022-01-12

**Authors:** Josiemer Mattei, Martha Tamez, June O’Neill, Sebastien Haneuse, Sigrid Mendoza, Jonathan Orozco, Andrea Lopez-Cepero, Carlos F. Ríos-Bedoya, Luis M. Falcón, Katherine L. Tucker, José F. Rodríguez-Orengo

**Affiliations:** 1 Department of Nutrition, Harvard T.H. Chan School of Public Health, Boston, Massachusetts; 2 Department of Biostatistics, Harvard T.H. Chan School of Public Health, Boston, Massachusetts; 3 FDI Clinical Research of Puerto Rico, San Juan; 4 McLaren Health Care, Graduate Medical Education, Grand Blanc, Michigan; 5 College of Fine Arts, Humanities and Social Sciences, University of Massachusetts, Lowell; 6 Department of Biomedical and Nutritional Sciences and Center for Population Health, University of Massachusetts, Lowell; 7 Department of Biochemistry, University of Puerto Rico Medical Sciences Campus, San Juan

## Abstract

**Importance:**

As public health emergencies become more prevalent, it is crucial to identify adverse physical and mental health conditions that may be triggered by natural disasters. There is a lack of data on whether Hurricane Maria in 2017 influenced the disease burden of adults in Puerto Rico.

**Objective:**

To estimate the prevalence of chronic diseases and their associated risk factors among adults living in Puerto Rico before and after Hurricane Maria in 2017.

**Design, Setting, and Participants:**

This cross-sectional study used data from 2 previous cross-sectional studies, including the pre–Hurricane Maria Puerto Rico Assessment on Diet, Lifestyles and Disease (PRADLAD) study, conducted in 2015, and the post–Hurricane Maria Puerto Rico Observational Study of Psychosocial, Environmental, and Chronic Disease Trends (PROSPECT), conducted in 2019. Participants included adults aged 30 to 75 years residing in Puerto Rico. Data were analyzed from April to October 2020.

**Exposures:**

Self-reported data were obtained on sociodemographic, lifestyle, and psychosocial factors and medically diagnosed conditions using validated questionnaires. Anthropometrics were measured in triplicate.

**Main Outcomes and Measures:**

Data were obtained using similar protocols in both studies. Characteristics were contrasted for all participants across studies and for 87 PRADLAD participants who returned to PROSPECT.

**Results:**

A total of 825 participants from both cohorts were included, with 380 PRADLAD participants and 532 PROSPECT participants. In the 2019 PROSPECT study, the mean (SD) age was 53.7 (10.8) years, and 363 participants (68.2%) were assigned female at birth and 169 participants (31.8%) were assigned male at birth. In the 2019 cohort, 360 participants (67.7%) had college education or higher, 205 participants (38.5%) reported annual income greater than $20 001, and 263 participants (49.5%) were employed. Most sociodemographic variables were similar between studies, except for higher income and employment after the hurricane. In the main analysis, participants in 2019, compared with participants in 2015, had higher abdominal obesity (389 participants [73.2%] vs 233 participants [61.3%]), sedentarism (236 participants [44.4%] vs 136 participants [35.8%]), binge drinking (95 participants [17.9%] vs 46 participants [12.1%]), and social support (mean [SD] score, 26.9 [7.2] vs 24.7 [7.1]) but lower depressive symptoms (169 participants [31.7%] vs 200 participants [52.6%]) and perceived stress (mean [SD] score, 19.3 [9.5] vs 21.7 [7.7]). In 2019, compared with 2015, there were higher rates of hypertension (252 participants [47.3%] vs 149 participants [39.2%]), arthritis (172 participants [32.3%] vs 97 participants [25.6%]), high cholesterol (194 participants [36.4%] vs 90 participants [23.8%]), high triglycerides (123 participants [23.1%] vs 56 participants [14.7%]), eye disease (94 participants [17.6%] vs 48 participants [12.7%]), fatty liver disease (68 participants [12.8%] vs 29 participants [7.5%]), and osteoporosis (74 participants [13.9%] vs 20 participants [5.2%]). Secondary analysis for the 87 returning participants showed similar results.

**Conclusions and Relevance:**

In this cross-sectional study, a higher prevalence of unhealthy behaviors and chronic conditions was noted among adults in Puerto Rico after Hurricane Maria, warranting long-term studies. Psychosocial factors were better, but still need attention. As natural disasters intensify, efforts should focus on continuous surveillance of health outcomes and promoting healthy behaviors, positive emotional health, and disease control, particularly in populations with higher risk for poor health.

## Key Points

**Question:**

What was the prevalence of chronic diseases and their associated risk factors among adults living in Puerto Rico before and after Hurricane Maria?

**Findings:**

This cross-sectional study using data from 825 participants in 2 studies conducted in 2015 and 2019 found statistically significantly higher prevalence of unhealthy lifestyle behaviors and multiple chronic conditions among adults in Puerto Rico after Hurricane Maria. Higher social support, lower depressive symptoms, and lower perceived stress were observed after the hurricane.

**Meaning:**

These findings suggest that as public health emergencies upsurge, continuous efforts will be necessary to sustain healthy behaviors, positive emotional health, and low rates of chronic diseases.

## Teaser

This cross-sectional study assesses the prevalence of chronic diseases and their associated risk factors among adults living in Puerto Rico before vs after Hurricane Maria.

## Introduction

In September 2017, the US territory of Puerto Rico was hit coast-to-coast by the exceptionally destructive category-4 Hurricane Maria.^1^ The damages to the infrastructure were massive and widespread, leaving residents without transport, power, or communication. There were severe limitations in access to basic needs, such as food, potable water, and health services, and an excessive death toll was reported.^2,3,4^

Exposures to natural disasters have been associated with adverse mental and physical health. For example, 1 year after Hurricane Katrina in New Orleans, Louisiana, which made landfall in 2005, more than half of a sample of individuals who had evacuated the area met the criteria for posttraumatic stress disorder (PTSD) and most had depression and anxiety.^5^ Twelve years later, 1 in 6 mothers with low income who endured the hurricane still had symptoms indicative of PTSD, and psychological distress remained high.^6^ Data from patients with acute myocardial infarction found a more than a 3-fold increase in admissions for subsequent attacks during the 6 years after Hurricane Katrina; these were accompanied by higher rates of psychiatric comorbidities, smoking, lack of health insurance, and unemployment, compared with rates before Hurricane Katrina.^7^ Just 2 years after the 2011 earthquake in East Japan, the proportion of individuals with overweight, diabetes, hypertension, dyslipidemia, liver dysfunction, atrial fibrillation, and gastrointestinal diseases increased.^8,9^ Unhealthy lifestyle behaviors after disasters may mediate the direct adverse health consequences of disasters. For example, poor dietary intake was noted after the 2011 earthquake in Japan,^9^ and analysis of data from 10 natural disasters has found that 22% to 40% of individuals coped with postdisaster emotions by drinking alcohol.^10^

Despite this evidence, little is known about the role of Hurricane Maria on the prevalence of chronic diseases and their risk factors. Some small studies have suggested associations with adverse health outcomes. An online survey among residents of Puerto Rico who were displaced to Florida or other regions of Puerto Rico reported a high prevalence of PTSD and generalized anxiety disorder.^11^ Another survey found that 7.2% of youths reported clinically significant symptoms of PTSD 5 to 8 months after Hurricane Maria.^12^ Still, there are scarce data on behavioral and psychosocial risk factors of chronic conditions before vs after Hurricane Maria in Puerto Rico. This gap is concerning, as the adult population of Puerto Rico already had high rates of multiple chronic conditions and emotional distress in the years before Maria.^13,14,15^ In 2016, the Behavioral Risk Factor Surveillance System (BRFSS) reported a prevalence of 61.8% for overweight or obesity, 41.7% for physical inactivity, 23.8% for arthritis, 18.2% for depression, 15.3% for diabetes, 12.5% for binge drinking, 10.6% for tobacco use, and 7.8% for coronary heart disease.^13^ In response, this study aimed to estimate the prevalence of chronic diseases and their associated risk factors among adults living in Puerto Rico before and after Hurricane Maria, using data from 2 studies conducted in 2015 and 2019.

## Methods

This cross-sectional study was approved by the institutional review boards of Harvard T.H. Chan School of Public Health, Ponce Health Sciences University, and University of Massachusetts, Lowell. All participants provided written informed consent. We followed the Strengthening the Reporting of Observational Studies in Epidemiology (STROBE) reporting guideline for cross-sectional studies.

### Study Population

We used data from 2 studies conducted in Puerto Rico. The first study was the Puerto Rico Assessment on Diet, Lifestyles and Disease (PRADLAD) study.^14,16^ Conducted in 2015, PRADLAD was a cross-sectional survey of a convenience sample of adults recruited at 3 partner clinics in the San Juan metropolitan area of Puerto Rico; the metropolitan area comprises nearly two-thirds of the population. Individuals who expressed interest were screened for eligibility based on the following criteria: current residence in Puerto Rico for at least 10 months of the previous year, aged 30 to 75 years (when chronic disease risk factors typically start to emerge), and able to answer questions without assistance.

The second study was Puerto Rico Observational Study of Psychosocial, Environmental, and Chronic Disease Trends (PROSPECT), an ongoing prospective, population-based cohort study initiated in March 2019, recruiting adults living in all of Puerto Rico.^17^ Our analysis used baseline cross-sectional data only. Recruitment in PROSPECT was conducted using a multistaged approach by enumerating potentially eligible households using 2010 Census block frames with socioeconomic and demographic data then randomly inviting 1 participant per qualified household and by advertising at communitywide events and locations. Additionally, PRADLAD participants were invited. Eligibility criteria include aged 30 to 75 years, living in Puerto Rico at the time of enrollment and at least the previous year, not planning to move outside the island within 3 years, living in a stable dwelling, and able to answer questions without assistance. Eligible individuals were invited to a baseline in-person visit at 1 of 55 islandwide partner clinics to answer multiple questionnaires, obtain clinical measurements, and collect biological samples.

### Data Collection

Trained research assistants conducted all data collection in Spanish (English was available on request). Data collection procedures were similar for both studies. Information was obtained on age, assigned sex at birth, ethnicity, educational attainment, household income, marital status, work history, migration history, area of residence, household composition, health insurance status, and self-rated health. Ethnicity was included as a variable in this study because out-migration from Puerto Rico after Hurricane Maria was significant and sustained, according to the census data; thus, including this variable could indicate who was leaving the island. Participants were asked to self-report whether a health professional had diagnosed each of a comprehensive list of conditions and whether they currently had the condition.

Questions were included to assess food insufficiency and receiving the Puerto Rico Nutrition Assistance Program (NAP) or the Women and Infant and Children (WIC) food assistance program within the past 6 months. Detailed data on alcohol intake and smoking habits were assessed. The Paffenbarger questionnaire of the Harvard Alumni Activity Survey was used to assess the number of hours spent per day at various activity levels that were then multiplied by predefined weighting factors to derive a physical activity score; a lower score indicates more sedentary time using modified cutoffs for this population.^18,19^ Information on the amount of sleep (hours per day) and quality of sleep (insomnia and nonrestorative sleep) was assessed with a questionnaire previously used among residents of Puerto Rico.^20,21^ The research assistant measured waist and hip circumference twice following standardized protocols; the mean measure was used.

Perceived stress during the previous month was measured with a perceived stress scale that has shown good internal consistency in English- and Spanish-speaking Hispanic individuals.^22,23^ Depressive symptoms were captured using the Center for Epidemiologic Studies Depression Scale (CES-D), which had Cronbach α of 0.90 in a sample of older adults in Puerto Rico.^24,25,26^ Social support was assessed using the Interpersonal Support Evaluation List–12-item, which has demonstrated internal consistency, reliability, and convergent validity across languages and Hispanic ethnicities, including Puerto Rican individuals.^27,28,29^

From the original 380 PRADLAD participants, 358 consented to be contacted for future studies, of whom 338 were invited to be screened for PROSPECT (eFigure 1 in the Supplement). After exclusions owing to no response (110 participants [33%]), no interest (26 participants [8%]), or unavailable contact information (51 participants [15%]), 151 PRADLAD participants were screened for PROSPECT. Of these, 90 were found to be eligible, comprising a 69% response rate. After excluding 3 participants with unreliable interviews, 87 PRADLAD participants completed the PROSPECT baseline interview.

PROSPECT participants were recruited from 3 sources: the returning PRADLAD participants (87 participants), household enumeration (23 participants), and communitywide strategies (422 participants) (eFigure 2 in the Supplement). We report fewer individuals recruited through enumeration than through community strategies because enumeration requires more time to implement. However, response rates were similar for both approaches. Through these efforts, a total of 532 participants had completed the baseline PROSPECT interview as of March 16, 2020, the date at which the study paused owing to the COVID-19 pandemic. While PROSPECT resumed in August 2020, the cutoff date was adopted in this study to remove potential behavioral and psychosocial changes during the pandemic.

### Statistical Analysis

Differences in characteristics between PRADLAD and PROSPECT participants were assessed using χ^2^ tests for categorical variables and *t* tests for continuous variables. Analysis was conducted for the complete study population of PRADLAD (380 participants) and the sample of PROSPECT (532 participants), and for the subset of 87 participants who were enrolled in both PRADLAD and PROSPECT (hereafter referred to as *returning participants*). In supplemental analysis, general linear models were used to estimate age-standardized characteristics using the age-adjustment weights of the 2010 mainland US population to consider potential differences in age distribution between studies. Additionally, we compared characteristics for PRADLAD vs PROSPECT participants excluding the returning participants, and for returning participants vs PRADLAD participants who did not participate in PROSPECT. All analyses were conducted using SAS statistical software version 9.4 (SAS Institute Inc). Significant differences were considered at a 2-tailed *P* < .05. Data were analyzed from April to October 2020.

## Results

A total of 825 participants from both cohorts were included, with 380 participants in the 2015 PRADLAD study and 532 participants in the 2019 PROSPECT study, including 87 participants returning from the 2015 study. In the 2015 study, the mean (SD) age was 51.5 (11.2) years, 249 participants (65.5%) were assigned female at birth and 131 participants (34.5%) were assigned male at birth, and 60 participants (15.9%) lived in rural areas (Table 1). Compared with PRADLAD participants, the participants from the PROSPECT sample in 2019 were older (mean [SD] age, 53.7 10.8] years), more likely to report Puerto Rican ethnicity (491 participants [92.3%] vs 310 participants [81.6%]), have household income higher than $20 001 per year (205 participants [38.5%] vs 72 participants [18.9%]), be currently employed (263 participants [49.5%] vs 139 participants [36.5%]), and have lived in Puerto Rico most of their lives (505 participants [94.9%] vs 337 participants [88.6%]), but less likely to be single (137 participants [25.8%] vs 138 participants [36.3%]) and to plan moving from Puerto Rico (29 participants [5.5%] vs 67 participants [17.6%]). In the 2019 cohort, 363 participants (68.2%) were assigned female at birth and 169 participants (31.8%) were assigned male at birth. Similar differences were noted when contrasting the returning participants from PRADLAD in 2015 vs 2019 (Table 2). Additionally, returning participants were more likely to report receiving NAP in 2019 vs 2015 (48 participants [55.1%] vs 35 participants [40.2%]). When we excluded returning participants from analysis, results remained similar, except for higher educational attainment among participants of PROSPECT vs PRADLAD (74 participants [16.6%] vs 29 participants [9.9%]) (eTable 1 in the Supplement).

**Table 1.** Sociodemographic Characteristics of Adult Participants of the 2015 PRADLAD Study and the 2019 PROSPECT Study

Characteristic	Participants, No. (%)		*P* value
	PRADLAD 2015 (n = 380)	PROSPECT 2019 (n = 532)	
Age, mean (SD), y	51.5 (11.2)	53.7 (10.8)	<.001
Sex assigned at birth			
Female	249 (65.5)	363 (68.2)	.39
Male	131 (34.5)	169 (31.8)	
Rural area of residence	60 (15.9)	106 (19.9)	.12
Puerto Rican ethnicity	310 (81.6)	491 (92.3)	<.001
Marital status			
Married or living with partner	163 (42.8)	256 (48.1)	.003
Divorced, separated, or widowed	79 (20.9)	139 (26.1)	
Single	138 (36.3)	137 (25.8)	
Education			
<8th grade	45 (11.9)	28 (5.2)	.50
9th-12th grade or GED	113 (29.8)	144 (27.1)	
Some college or college degree	181 (47.7)	278 (52.3)	
Graduate school	40 (10.6)	82 (15.4)	
Household income, $			
0-10 000	228 (59.9)	191 (35.9)	<.001
10 001-20 000	81 (21.2)	136 (25.6)	
>20 001	72 (18.9)	205 (38.5)	
Employment			
Currently employed	139 (36.5)	263 (49.5)	.001
Retired or stay-at-home	183 (48.2)	212 (39.8)	
Unemployed	58 (15.3)	57 (10.7)	
Health insurance			
Government-assisted	211 (55.4)	195 (36.6)	<.001
Private	141 (37.0)	309 (58.1)	
Uninsured	29 (7.6)	28 (5.3)	
Food security and assistance			
Frequent food insufficiency	55 (14.5)	64 (12.1)	.29
WIC food assistance	26 (6.8)	30 (5.7)	.46
NAP	194 (51.1)	288 (54.1)	.37
Migration history			
Lived in Puerto Rico most of their life	337 (88.6)	505 (94.9)	<.001
Lived in mainland US ≥1 y	106 (27.8)	138 (26.0)	.55
Plans to move from Puerto Rico	67 (17.6)	29 (5.5)	<.001

Abbreviations: NAP, Puerto Rico Nutrition Assistance Program; PRADLAD, Puerto Rico Assessment on Diet, Lifestyles and Disease; PROSPECT, Puerto Rico Observational Study of Psychosocial, Environmental, and Chronic Disease Trends; WIC, Women and Infant and Children.

**Table 2.** Sociodemographic Characteristics of Adult Participants of the 2015 PRADLAD Study Who Were Also Recruited Into the 2019 PROSPECT Study

Characteristic	PRADLAD participants, %		*P* value
	2015 (n = 87)	2019 (n = 87)	
Age, mean (SD), y	52.8 (10.5)	56.4 (10.7)	.03
Sex assigned at birth			
Female	62 (71.3)	62 (71.3)	>.99
Male	25 (28.7)	25 (28.7)	
Rural area of residence	10 (11.5)	9 (10.3)	.80
Puerto Rican ethnicity	73 (83.9)	73 (83.9)	>.99
Marital status			
Married or living with partner	39 (44.8)	37 (42.5)	.045
Divorced, separated, or widowed	15 (17.3)	28 (32.2)	
Single	33 (37.9)	22 (25.3)	
Education			
<8th grade	5 (5.8)	6 (6.9)	.63
9th-12th grade or GED	28 (32.6)	25 (28.7)	
Some college or college degree	42 (48.8)	48 (55.2)	
Graduate school	11 (12.8)	8 (9.2)	
Household income, $			
0-10 000	51 (58.4)	36 (41.8)	.08
10 001-20 000	15 (16.9)	25 (29.1)	
>20 001	21 (24.7)	25 (29.1)	
Employment			
Currently employed	31 (35.6)	41 (47.1)	.97
Retired or stay-at-home	47 (54.0)	32 (36.4)	
Unemployed	9 (10.4)	14 (16.5)	
Health insurance			
Government-assisted	44 (50.7)	43 (49.4)	.92
Private	37 (42.4)	39 (44.8)	
Uninsured	6 (6.9)	5 (5.8)	
Food security and assistance			
Frequent food insufficiency	10 (11.5)	12 (13.4)	.70
WIC food assistance	2 (2.3)	4 (4.3)	.46
NAP	35 (40.2)	48 (55.1)	.049
Migration history			
Lived in Puerto Rico most of their life	78 (89.7)	81 (93.1)	.42
Lived in mainland US ≥1 y	16 (18.4)	21 (24.1)	.36
Plans to move from Puerto Rico	11 (12.8)	3 (3.5)	.03

Abbreviations: NAP, Puerto Rico Nutrition Assistance Program; PRADLAD, Puerto Rico Assessment on Diet, Lifestyles and Disease; WIC, Women and Infant and Children.

PROSPECT participants, compared with all PRADLAD participants, had higher prevalence of abdominal obesity (389 participants [73.2%] vs 233 participants [61.3%]), high waist-to-hip ratio (443 participants [83.2%] vs 292 participants [76.8%]), sedentary physical activity (236 participants [44.4%] vs 136 participants [35.8%]), alcohol use (255 participants [47.9%] vs 99 participants [26.1%]), binge drinking (95 participants [17.9%] vs 46 participants [12.1%]), and yearly influenza vaccination (169 participants [31.8%] vs 96 participants [25.3%]), but lower self-rated health as poor or fair (179 participants [33.7%] vs 152 participants [40.0%]) (Table 3). PROSPECT participants, compared with PRADLAD participants, also reported lower mean depressive symptoms score (mean [SD] score, 13.1 [11.8] vs 17.6 [12.6]), lower prevalence of depressive symptoms (169 participants [31.7%] vs 200 participants [52.6%]), and lower perceived stress score (mean [SD] score, 19.3 [9.5] vs 21.7 [7.7]) but higher mean social support score (mean [SD] score, 26.9 [7.2] vs 24.7 [7.1]). Differences were noted for 2019 PROSPECT participants compared with 2015 PRADLAD participants for higher hypertension (252 participants [47.3%] vs 149 participants [39.2%]), arthritis (172 participants [32.3%] vs 97 participants [25.6%]), high cholesterol (194 participants [36.4%] vs 90 participants [23.8%]), high triglycerides (123 participants [23.1%] vs 56 participants [14.7%]), eye disease (94 participants [17.6%] vs 48 participants [12.7%]), fatty liver disease (68 participants [12.8%] vs 29 participants [7.5%]), and osteoporosis (74 participants [13.9%] vs 20 participants [5.2%]) (Figure; eTable 2 in the Supplement). In 2019 vs 2015, use of medication was significantly higher for high cholesterol (126 participants [23.7%] vs 56 participants [14.7%]), high triglycerides (63 participants [11.8%] vs 23 participants [6.1%]), respiratory problems (93 participants [17.5%] vs 42 participants [11.1%]), and osteoporosis (40 participants [7.5%] vs 8 participants [2.1%]); no other significant differences in medication use were detected.

**Table 3.** Lifestyle and Psychosocial Risk Factors of Adult Participants of the 2015 PRADLAD Study and the 2019 PROSPECT Cohort

Factor	Participants, No. (%)		*P* value
	PRADLAD 2015 (n = 380)	PROSPECT 2019 (n = 532)	
Lifestyle factors			
Abdominal obesity	233 (61.3)	389 (73.2)	<.001
High waist-to-hip ratio	292 (76.8)	443 (83.2)	.02
Self-rated poor or fair health	152 (40)	179 (33.7)	.049
Self-rated poor or fair dietary habits	116 (30.5)	163 (30.6)	.97
Sedentary physical activity	136 (35.8)	236 (44.4)	.009
Current smoker	68 (17.9)	77 (14.5)	.16
Current alcohol drinker	99 (26.1)	255 (47.9)	<.001
Binge drinker	46 (12.1)	95 (17.9)	.02
Recurrent sleeping difficulties	81 (21.3)	132 (24.8)	.22
Extreme sleeping hours (<7 h or >9 h)	193 (50.7)	253 (47.5)	.35
Yearly influenza vaccination	96 (25.3)	169 (31.8)	.03
Psychosocial factors, mean (SD)			
Depressive symptoms score	17.6 (12.6)	13.1 (11.8)	<.001
Depression-like symptoms (score >16), No. (%)	200 (52.6)	169 (31.7)	<.001
Perceived stress score	21.7 (7.7)	19.3 (9.5)	<.001
Social support score			
Total	24.7 (7.1)	26.9 (7.2)	<.001
Appraisal	8.4 (2.8)	9.3 (2.8)	<.001
Belonging	8.2 (2.8)	8.8 (3.0)	.001
Tangible	8.0 (2.6)	8.6 (2.9)	<.001

Abbreviations: PRADLAD, Puerto Rico Assessment on Diet, Lifestyles and Disease; PROSPECT, Puerto Rico Observational Study of Psychosocial, Environmental, and Chronic Disease Trends.

**Figure.**
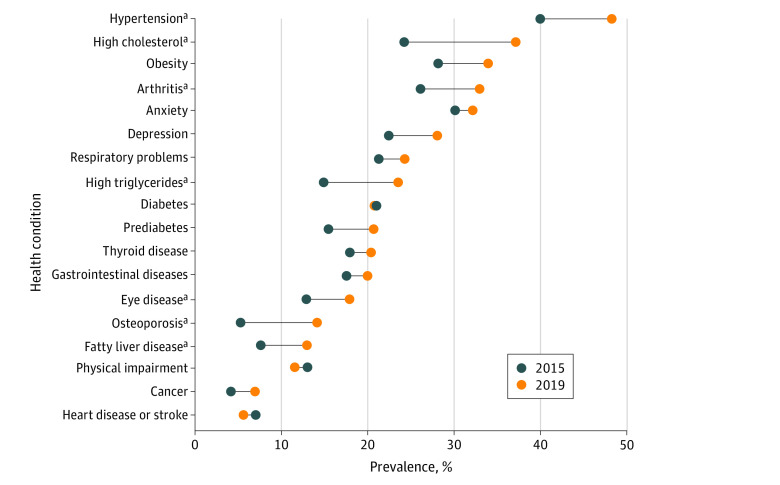
Chronic Diseases Reported by Adults in 2015, Before Hurricane Maria, and 2019, After Hurricane Maria The 2015 study included 380 participants and the 2019 study included 532 participants. ^a^Significantly different at *P* < .05. Differences assessed using χ^2^ test.

Similar results were observed for these comparisons using age-standardization, although differences were attenuated (eTable 3 in the Supplement). Results were also similar when excluding the PRADLAD returning participants, except that more prediabetes was noted in 2019 vs 2015 (eTable 4 in the Supplement).

In the subset of returning participants, differences in the prevalence of lifestyle risk factors and chronic conditions remained in the same direction as those observed when contrasting the full studies, although some differences were weakened (Table 4). Within this subset, more participants reported gastrointestinal disease in 2019 than 2015 (27 participants [30.6%] vs 13 participants [15.1%]) and heart disease or stroke (8 participants [9.5%] vs 2 participants [2.3%]). Age-standardized results were similar, albeit attenuated (eTable 5 in the Supplement).

**Table 4.** Lifestyle and Psychosocial Risk Factors and Self-reported Chronic Conditions of Adult Participants of the 2015 PRADLAD Study and Again in the Subsequent 2019 PROSPECT Study

Factor	PRADLAD Participants, No. (%)		*P* value
	2015 (n = 87)	2019 (n = 87)	
Lifestyle factors			
Abdominal obesity	56 (64.5)	65 (74.7)	.16
High waist-to-hip ratio	73 (83.8)	67 (77.1)	.29
Self-rated poor or fair health	30 (34.5)	33 (37.9)	.64
Self-rated poor or fair dietary habits	30 (34.5)	27 (31.0)	.63
Sedentary physical activity	23 (26.4)	25 (28.7)	.73
Current smoker	14 (16.1)	17 (19.5)	.55
Current alcohol drinker	28 (31.0)	33 (37.9)	.34
Binge drinker	13 (14.9)	16 (18.4)	.66
Recurrent sleeping difficulties	20 (23.0)	23 (26.4)	.60
Extreme sleeping hours (<7 h or >9 h)	44 (50.0)	45 (51.8)	.81
Yearly influenza vaccination	30 (34.5)	32 (36.8)	.75
Psychosocial factors, mean (SD)			
Depressive symptoms score	14.2 (12.2)	11.0 (9.9)	.06
Depression-like symptoms (score, >16), %	32 (36.4)	18 (20.8)	.03
Perceived stress score	20.3 (7.7)	17.9 (8.3)	.04
Social support score			
Total	26.7 (6.8)	27.8 (6.3)	.30
Appraisal	8.8 (2.7)	9.9 (2.2)	.003
Belonging	9.1 (2.7)	8.8 (2.9)	.49
Tangible	8.7 (2.4)	8.9 (2.6)	.51
Self-reported medical diagnoses			
Hypertension	35 (40.0)	40 (46.4)	.40
Anxiety	25 (28.6)	31 (36.0)	.30
Obesity	30 (34.1)	37 (42.7)	.26
Arthritis	23 (26.7)	29 (33.3)	.35
High cholesterol	17 (19.1)	35 (40.2)	.003
Depression	16 (18.8)	25 (29.3)	.11
Respiratory problems	20 (23.5)	24 (27.9)	.51
Diabetes	20 (23.3)	25 (28.6)	.43
Thyroid disease	16 (18.8)	18 (20.5)	.79
Gastrointestinal diseases	13 (15.1)	27 (30.6)	.02
Prediabetes	18 (20.9)	20 (23.5)	.68
High triglycerides	14 (16.1)	22 (25.0)	.16
Eye disease	15 (16.7)	21 (24.7)	.20
Physical impairment	14 (16.5)	12 (14.1)	.67
Heart disease/stroke	2 (2.3)	8 (9.5)	.046
Cancer	4 (4.8)	6 (6.9)	.55
Fatty liver disease	6 (7.4)	17 (19.7)	.02
Osteoporosis	6 (7.0)	14 (15.7)	.07

Abbreviation: PRADLAD, Puerto Rico Assessment on Diet, Lifestyles and Disease.

We conducted some sensitivity analyses. First, we contrasted PRADLAD participants returning to PROSPECT with those who did not participate in PROSPECT. Sociodemographic characteristics were similar, except that more returning participants vs nonreturning participants reported living alone and fewer reported receiving NAP or having lived in the mainland US for at least 1 year (eTable 6 in the Supplement). Health conditions and risk factors were also similar between these subgroups, except that returning participants vs nonreturning participants had lower depressive symptom scores and higher social support scores (eTable 7 in the Supplement). We also compared 2019 sociodemographic data from the 87 participants returning from PRADLAD to 2019 data from the rest of the PROSPECT participants; similar characteristics were noted except for age, rural residency, Puerto Rican ethnicity, and health insurance type (eTable 8 in the Supplement).

## Discussion

In this cross-sectional study using data from 2 studies before vs after Hurricane Maria (ie, 2015 vs 2019), we found a higher prevalence of unhealthy behaviors and several chronic conditions and a more positive, albeit still concerning, social-emotional health profile after the hurricane. These observations were generally corroborated in a subset of individuals participating in both studies, although some results were attenuated, likely owing to the small sample size. The results contribute new evidence on the excessive burden of multiple chronic disease after a massive natural disaster, helping expand the existing literature that has been mostly limited to postdisaster reports and to communicable diseases.^30,31^

The 2019 PROSPECT participants had similar characteristics to the 2015 PRADLAD participants, although the older age in the 2019 sample may contribute to higher prevalence of some chronic conditions. Also, income and employment rates were higher in 2019. These observations are supported by data from the US Bureau of Labor Statistics.^32^ However, it is possible that instead of socioeconomic improvement, the differences are a product of the larger impact of the hurricane on people living with low incomes or unemployment, who were more prone to experience damages but received less assistance and were more likely to leave the island.^33^ Also, PRADLAD participants with a lower socioeconomic profile may have had a strong migratory intent, hastened by Hurricane Maria. Furthermore, posthurricane mortality was highest for individuals living in municipalities with the lowest socioeconomic status.^2^ Stable employment and income after the hurricane may have contributed to the observed healthier psychosocial profile. Additionally, higher private health insurance enrollment after the hurricane could translate to more chronic diseases being diagnosed.

Other noted differences, such as a higher percentage of Puerto Rican individuals (vs other ethnicities) and fewer single individuals, might reflect specific recruitment strategies, such as having 1 of the recruitment sites in the 2015 study within a community of individuals from the Dominican Republic. Interestingly, participants in 2019 were more likely to report that they had lived in Puerto Rico their whole lives. A survey among Latino immigrants to the mainland US showed that 84% of Puerto Rican individuals had been to the mainland US at least once.^34^ While posthurricane migration was substantial,^35,36^ it may be possible that lack of previous connections to the mainland US, or a strong cultural and social attachment to the island, discouraged some adults from moving.

In 2019, several unhealthy lifestyle factors became more prevalent. These observations align with BRFSS data from Puerto Rico showing higher age-adjusted prevalence of body mass index–classified overweight and obesity in 2019 (68.8%) compared with 2015 (66.1%).^13^ In 2015, 47.1% of adults reported not participating in any exercise and 13.7% reported binge drinking, compared with 49.8% reporting not participating in exercise and 14.1% reporting binge drinking by 2019.^13^ In addition to these factors, we observed higher prevalence of hypertension, arthritis, and high cholesterol after the hurricane, but BRFSS reported decreases in these conditions from 2015 to 2019. Discrepancies may be due to methodological differences. Notably, our results remained similar after age-standardization, diminishing the possibility of shifts in age structure as a discerning factor. Other conditions for which we observed higher prevalence in 2019, and which are not tracked by the BRFSS, include high triglycerides, eye disease, fatty liver disease, and osteoporosis.

Interestingly, there were no significant differences in the prevalence of diabetes, physical impairment, or heart disease or stroke. People with chronic conditions often require considerable medical care, such as multiple medications, specialized services or medical devices, and frequent visits to diverse medical specialists. Studies in Puerto Rico documented the severe posthurricane disruptions to these medical services, as well as widespread physical destruction to health care facilities or shortages in electricity, communications, water, and transportation, requiring many individuals with chronic conditions to leave the island for essential medical care.^33,37,38,39,40,41^ Many clinical workers also left the island.^38^ For people with physical disabilities, the broken infrastructure exacerbated already existing vulnerabilities.^42^ Notably, heart disease and diabetes were among the top causes for excess deaths after Hurricane Maria,^3^ which may further explain the lack of differences in these conditions. Within the subset of the returning participants, 3-fold higher heart diseases or stroke was observed in 2019. It may be possible that unhealthy behaviors and higher biological risk factors hastened heart conditions in these adults. Gastrointestinal disease was twice as high in 2019 in the subset, consistent with another posthurricane report from the island.^39^

From 2015 to 2019, we noted lower depressive symptom and stress scores but higher social support scores. Mental and emotional health are usually worsened after natural disasters, yet studies also report increases in protective factors, including resilience and coping strategies.^43,44,45^ In a study conducted after several storms in Mexico, depression symptoms remained high compared with prestorm periods, yet social support returned to better than prestorm levels in some communities.^46^ Among survivors of major bushfires, depression risk was higher for individuals with fewer social connections, connected to other people with depression, or connected to people who had left their community.^47^ Optimism, social support, and social ties are strong among Puerto Rican adults and have been shown to have a protective association against psychological distress in the face of stressful life events.^29,33,48^ Sustained high-quality mental health care, breaking cultural barriers for seeking support, and continued compassionate and culturally relevant communal support may strengthen coping and resiliency among disaster survivors.^49,50^ These strategies are essential, given that nearly one-third of adults exhibited depressive symptoms in 2019.

Social determinants of health, namely poverty, the physical environment, environmental factors, and shortages in safe food and water, may contribute to postdisaster vulnerability to the adverse health outcomes reported here.^51^ Hurricane Maria revealed underlying social and structural inequities, including discriminatory practices, systemic racism, human rights violations, negligence, and ineffectual policies, that led to inadequate emergency responses.^52,53^ Analysis of federal spending estimates showed that the federal government responded faster and better to Hurricanes Harvey and Irma in Texas and Florida, compared with Hurricane Maria in Puerto Rico, a variation that was not commensurate with storm severity and need after landfall.^54^ Similar disparities have been reported after Hurricane Katrina, where cardiovascular disease rates between Black and White older adults were exacerbated during and following landfall; after the Haiti 2010 earthquake, where women experienced more survival needs, violence, exploitation, and class- and race-based stigmatization than men; and after the September 11, 2001, terrorist attacks in New York, New York, where socially or economically marginalized individuals had higher depression than those who were more socially and financially secure.^55,56,57^

### Limitations

This study has some limitations. First, we used data from 2 different studies. However, the results were corroborated in a subset of individuals followed from 2015 to 2019. Second, the recruitment strategies, including recruiting in clinics in the San Juan metropolitan area only in the 2015 study, may limit representation of the general Puerto Rico population. This limitation is lessened by the socioeconomic and health profile representation of PRADLAD participants and by the islandwide efforts in PROSPECT—approximately 35% of the PROSPECT sample used in this analysis was recruited from the nonmetropolitan area.^16,17^ Furthermore, the sociodemographic characteristics of the returning participants matched population-wide surveillance data, and there were few differences noted in the characteristics of returning participants vs those whom we could not contact. Using mainland US-based census data for age-standardized estimates may not fully represent the age structure of Puerto Rico; however, sampling weights for Puerto Rico were unavailable. Fourth, although behavioral and psychosocial risk factors were measured using validated tools, chronic disease status was self-reported and not validated against objective measures or medical records. The widespread health insurance coverage in Puerto Rico may facilitate awareness of diagnoses among participants, lessening this concern. Any underreporting of conditions would likely be similar for both studies, which may attenuate our estimates but still support the main conclusions. Fifth, we cannot distinguish if the observed differences were owing to ongoing trends or directly triggered by Hurricane Maria. However, existing evidence of similar increases in chronic conditions after natural disasters supports that hurricanes and other disasters may trigger or exacerbate disease state.^58,59^ Notably, we restricted the PROSPECT sample to individuals recruited before COVID-19 to avoid potential changes in health conditions and risk factors during the pandemic. Future studies should analyze the compounding effect of concurrent public health emergencies on the health of Puerto Rico residents.

## Conclusions

The finds of this cross-sectional study suggest that, given the observed high prevalence of several risk factors and chronic disease after Hurricane Maria, clinical and public health prevention measures should be prioritized in disaster-prone areas. Health promotion strategies should be strengthened to sustain a culture of health at all times. It is essential to implement populationwide surveillance systems on chronic diseases and their risk factors for continued monitoring, especially to detect changes during unforeseen public health emergencies. Relevant policies that could help curb detrimental health impacts after natural disasters include strengthening the health care system, developing policies that address social determinants of health and social inequities, strengthening social services and nutrition assistance programs (including emergency funds), enhancing food and medical stockpiles, and investing in climate resiliency, emergency preparedness, and disaster response.^39,60^ Lastly, public health and clinical programs should leverage positive psychosocial factors for better postdisaster socioemotional health. Timely attention to these issues is imperative, given that natural disasters are becoming more prevalent, destructive, and costly,^61^ particularly in populations already at high risk of poor health,^58^ and their impact on health persists for years.^62^

## Notes

Supplement. **eTable 1.** Sociodemographic Characteristics of Adult Participants of the 2015 PRADLAD Study and the 2019 PROSPECT Study, Excluding 87 Repeat Participants **eTable 2.** Self-reported Health Conditions of Adult Participants of the 2015 PRADLAD Study and the 2019 PROSPECT Study **eTable 3.** Age-Standardized Prevalence and Age-Standardized Means of Lifestyle and Psychosocial Risk Factors and Health Indicators of Adult Participants of the 2015 PRADLAD Study and the 2019 PROSPECT Study **eTable 4.** Lifestyle and Psychosocial Risk Factors and Health Indicators of Adult Participants of the 2015 PRADLAD Study and the 2019 PROSPECT Study **eTable 5.** Age-Standardized Prevalence and Age-Standardized Means of Lifestyle and Psychosocial Risk Factors and Health Indicators of Adult Participants of the 2015 PRADLAD Study and Again in the Subsequent 2019 PROSPECT Study **eTable 6.** Sociodemographic Characteristics of Adult Participants in the 2015 PRADLAD Study in Puerto Rico by Participation Status in the Subsequent PROSPECT Study **eTable 7.** Lifestyle and Psychosocial Risk Factors and Health Indicators of Adult Participants in the 2015 PRADLAD Study in Puerto Rico by Participation Status in the Subsequent PROSPECT Study **eTable 8.** Sociodemographic Characteristics of Adult Participants of the PRADLAD Study in 2019 and of PROSPECT Participants in 2019 Recruited Through Other Strategies (Not PRADLAD) **eFigure 1.** Flowchart of 2015 PRADLAD Participants Who Were Recontacted and Eligible to Participate in the 2019 PROSPECT Study **eFigure 2.** Flowchart of Recruitment of 2019 PROSPECT Participants as of March 16, 2020
