# Supplementary material for: Chronic Diseases and Associated Risk Factors Among Adults in Puerto Rico After Hurricane Maria
Source: JAMA Netw Open. 2022 Jan 12;5(1):e2139986. doi: 10.1001/jamanetworkopen.2021.39986 (PMC8756309; doi:10.1001/jamanetworkopen.2021.39986)
Supplement: Supplement. — eTable 1. Sociodemographic Characteristics of Adult Participants of the 2015 PRADLAD Study and the 2019 PROSPECT Study, Excluding 87 Repeat Participants eTable 2. Self-reported Health Conditions of Adult Participants of the 2015 PRADLAD Study and the 2019 PROSPECT Study eTable 3. Age-Standardized Prevalence and Age-Standardized Means of Lifestyle and Psychosocial Risk Factors and Health Indicators of Adult Participants of the 2015 PRADLAD Study and the 2019 PROSPECT Study eTable 4. Lifestyle and Psychosocial Risk Factors and Health Indicators of Adult Participants of the 2015 PRADLAD Study and the 2019 PROSPECT Study eTable 5. Age-Standardized Prevalence and Age-Standardized Means of Lifestyle and Psychosocial Risk Factors and Health Indicators of Adult Participants of the 2015 PRADLAD Study and Again in the Subsequent 2019 PROSPECT Study eTable 6. Sociodemographic Characteristics of Adult Participants in the 2015 PRADLAD Study in Puerto Rico by Participation Status in the Subsequent PROSPECT Study eTable 7. Lifestyle and Psychosocial Risk Factors and Health Indicators of Adult Participants in the 2015 PRADLAD Study in Puerto Rico by Participation Status in the Subsequent PROSPECT Study eTable 8. Sociodemographic Characteristics of Adult Participants of the PRADLAD Study in 2019 and of PROSPECT Participants in 2019 Recruited Through Other Strategies (Not PRADLAD) eFigure 1. Flowchart of 2015 PRADLAD Participants Who Were Recontacted and Eligible to Participate in the 2019 PROSPECT Study eFigure 2. Flowchart of Recruitment of 2019 PROSPECT Participants as of March 16, 2020 [file jamanetwopen-e2139986-s001.pdf]

Supplemental Online Content

Mattei J, Tamez M, O'Neill J, et al. Chronic diseases and associated risk factors among adults in Puerto Rico after Hurricane Maria. *JAMA Netw Open*. 2022;5(1):e2139986. doi:10.1001/jamanetworkopen.2021.39986

eTable 1. Sociodemographic Characteristics of Adult Participants of the 2015 PRADLAD Study and the 2019 PROSPECT Study, Excluding 87 Repeat Participants
eTable 2. Self-reported Health Conditions of Adult Participants of the 2015 PRADLAD Study and the 2019 PROSPECT Study
eTable 3. Age-Standardized Prevalence and Age-Standardized Means of Lifestyle and Psychosocial Risk Factors and Health Indicators of Adult Participants of the 2015 PRADLAD Study and the 2019 PROSPECT Study
eTable 4. Lifestyle and Psychosocial Risk Factors and Health Indicators of Adult Participants of the 2015 PRADLAD Study and the 2019 PROSPECT Study
eTable 5. Age-Standardized Prevalence and Age-Standardized Means of Lifestyle and Psychosocial Risk Factors and Health Indicators of Adult Participants of the 2015 PRADLAD Study and Again in the Subsequent 2019 PROSPECT Study
eTable 6. Sociodemographic Characteristics of Adult Participants in the 2015 PRADLAD Study in Puerto Rico by Participation Status in the Subsequent PROSPECT Study
eTable 7. Lifestyle and Psychosocial Risk Factors and Health Indicators of Adult Participants in the 2015 PRADLAD Study in Puerto Rico by Participation Status in the Subsequent PROSPECT Study
eTable 8. Sociodemographic Characteristics of Adult Participants of the PRADLAD Study in 2019 and of PROSPECT Participants in 2019 Recruited Through Other Strategies (Not PRADLAD)
eFigure 1. Flowchart of 2015 PRADLAD Participants Who Were Recontacted and Eligible to Participate in the 2019 PROSPECT Study
eFigure 2. Flowchart of Recruitment of 2019 PROSPECT Participants as of March 16, 2020

This supplemental material has been provided by the authors to give readers additional information about their work.

© 2022 Mattei J et al. *JAMA Network Open.*

eTable 1. Sociodemographic Characteristics of Adult Participants of the 2015 PRADLAD Study and the 2019 PROSPECT Study, Excluding 87 Repeat Participants

	**PRADLAD 2015 (n=293)**	**PROSPECT 2019 (n=447)**	***P*-value**
	**Percent or mean (SD)**	**Percent or mean (SD)**	
Age, years	51.1 (11.4)	53.2 (10.7)	0.012
Sex-at-birth			
Female	63.8	67.4	0.31
Male	36.2	32.6	
Rural area of residence	17.2	21.9	0.12
Puerto Rican ethnicity	80.9	93.8	<0.0001
Marital status			0.016
Married/living with partner	42.2	49.2	
Divorced/separated/widowed	22.0	24.9	
Single	35.8	25.8	
Educational attainment			<0.0001
Less than 8th grade	13.7	4.8	
9th–12th grade or GED	29.0	26.8	
Some college or college degree	47.4	51.8	
Graduate school	9.9	16.6	
Household income			<0.0001
$0–$10,000	60.4	34.8	
$10,001–$20,000	22.6	24.9	
> $20,001	17.0	40.3	
Employment			0.0005
Currently employed	36.9	50.0	
Retired/stay-at-home	46.4	40.4	
Unemployed	16.7	9.6	
Health insurance			0.18
Government-assisted	56.7	34.2	
Private	35.5	60.6	
Uninsured	7.8	5.2	
Food security and assistance			
Frequent food insufficiency	15.5	11.8	0.15
WIC food assistance	8.2	5.9	0.22
Government food assistance	54.4	53.5	0.81
Migration history			
Lived in PR most of their life	88.3	95.3	0.0004
Lived in mainland US at least 1 year	30.7	26.6	0.23
Plans to move from PR	19.1	5.9	<0.0001

© 2022 Mattei J et al. *JAMA Network Open.*

eTable 2. Self-reported Health Conditions of Adult Participants of the 2015 PRADLAD Study and the 2019 PROSPECT Study

	**PRADLAD 2015 (n=380)**	**PROSPECT 2019 (n=532)**	***P*-value**
Self-reported medical diagnoses; percent			
Hypertension	39.2	47.3	0.016
Anxiety	29.7	31.6	0.55
Obesity	27.7	33.3	0.08
Arthritis	25.6	32.3	0.014
High cholesterol	23.8	36.4	<0.0001
Depression	22.1	27.5	0.07
Respiratory problems	20.9	23.8	0.31
Diabetes	20.7	20.5	0.93
Thyroid disease	17.7	20.0	0.38
Gastrointestinal diseases	17.2	19.6	0.36
Pre-diabetes	15.2	20.3	0.05
High triglycerides	14.7	23.1	0.002
Eye disease	12.7	17.6	0.045
Physical impairment	12.7	11.3	0.54
Heart disease/stroke	6.8	5.5	0.43
Cancer	4.1	6.8	0.09
Fatty liver disease	7.5	12.8	0.013
Osteoporosis	5.2	13.9	<0.0001

© 2022 Mattei J et al. *JAMA Network Open.*

eTable 3. Age-Standardized Prevalence and Age-Standardized Means of Lifestyle and Psychosocial Risk Factors and Health Indicators of Adult Participants of the 2015 PRADLAD Study and the 2019 PROSPECT Study*

	**PRADLAD 2015 (n=380)**	**PROSPECT 2019 (n=532)**	***P*-value**
	**Percent or mean (SD)**	**Percent or mean (SD)**	
Lifestyle factors			
Abdominal obesity	59.5	70.2	0.001
High waist-to-hip ratio	74.4	80.2	0.038
Self-rated poor/fair health	38.5	30.1	0.009
Self-rated poor/fair dietary habits	32.7	32.7	0.98
Sedentary physical activity	36.2	41.3	0.12
Current smoker	15.4	16.8	0.63
Current alcohol drinker	29.3	47.2	<0.0001
Binge drinker	15.8	19.3	0.16
Recurrent sleeping difficulties	20.7	24.0	0.25
Extreme sleeping hours (<7h or >9h)	51.3	48.0	0.32
Yearly influenza vaccination	23.7	31.7	0.008
Psychosocial factors			
Depressive symptoms score	17.3 (0.79)	13.4 (0.64)	0.0002
Depression-likely symptoms (≥16)	51.0	32.2	<0.0001
Perceived stress score	21.9 (0.47)	19.4 (0.51)	0.001
Social support score	24.7 (0.44)	26.7 (0.39)	0.001
Appraisal	8.5 (0.17)	9.3 (0.15)	<0.0001
Belonging	8.2 (0.17)	8.7 (0.16)	0.016
Tangible	8.0 (0.16)	8.6 (0.15)	0.032
Self-reported medical diagnoses			
Hypertension	34.4	38.4	0.23
Anxiety	27.9	33.0	0.10
Obesity	26.4	32.4	0.05
Arthritis	22.8	25.5	0.48
Hypercholesterolemia	22.2	29.0	0.033
Depression	20.3	28.3	0.05
Respiratory problems	21.3	24.0	0.33
Diabetes	18.4	16.0	0.33
Thyroid disease	17.2	17.7	0.83
Gastrointestinal diseases	15.8	18.4	0.30
Pre-diabetes	14.0	17.1	0.20
Hypertriglyceridemia	13.1	18.8	0.024
Eye disease	11.6	13.4	0.43
Physical impairment	10.4	9.1	0.45
Heart disease/stroke	5.2	4.7	0.70
Cancer	3.7	5.0	0.94
Fatty liver disease	9.4	11.3	0.38
Osteoporosis	4.7	10.7	0.001

*Adjustment weights used to age-standardized estimates to the US 2010 population age distribution were as follows: ≤39 years=0.396579, 40-59 years=0. 0.371795, ≥60 years=0.231626. Based on the 2010 US census population data obtained from: US Census Bureau. 2010 US Census summary file 1 singe years of age and sex. Available from: http://factfinder2.census.gov/faces/tableservices/jsf/pages/productview.xhtml?pid=DEC_10_SF1_QTP2&prodType=table (accessed June 2, 2021).

© 2022 Mattei J et al. *JAMA Network Open.*

eTable 4. Lifestyle and Psychosocial Risk Factors and Health Indicators of Adult Participants of the 2015 PRADLAD Study and the 2019 PROSPECT Study

	**PRADLAD 2015 (n=293)**	**PROSPECT 2019 (n=447)**	***P*-value**
	**Percent or mean (SD)**	**Percent or mean (SD)**	
Lifestyle factors			
Abdominal obesity	60.3	72.9	0.001
High waist-to-hip ratio	74.6	84.4	0.002
Self-rated poor/fair health	41.8	32.8	0.013
Self-rated poor/fair dietary habits	29.5	30.6	0.75
Sedentary physical activity	45.9	47.4	0.71
Current smoker	19.2	13.5	0.041
Current alcohol drinker	25.4	49.9	<0.0001
Binge drinker	11.3	17.8	0.016
Recurrent sleeping difficulties	21.6	24.5	0.36
Extreme sleeping hours (<7h or >9h)	49.1	53.3	0.27
Yearly influenza vaccination	22.9	30.8	0.020
Psychosocial factors			
Depressive symptoms score	18.7 (12.5)	13.5 (12.1)	<0.0001
Depression-likely symptoms (>16)	57.5	33.7	<0.0001
Perceived stress score	22.2 (7.4)	19.6 (9.7)	0.0001
Social support score	24.1 (7.2)	26.7 (7.4)	<0.0001
Appraisal	8.3 (2.8)	9.2 (2.8)	<0.0001
Belonging	7.9 (2.7)	8.8 (3.1)	0.0001
Tangible	7.8 (2.6)	8.6 (2.9)	0.0001
Self-reported medical diagnoses			
Hypertension	39.0	47.5	0.025
Anxiety	30.0	30.7	0.85
Obesity	25.7	31.5	0.10
Arthritis	25.2	33.3	0.022
Hypercholesterolemia	25.3	35.7	0.003
Depression	23.1	27.2	0.21
Respiratory problems	20.1	23.0	0.36
Diabetes	20.0	19.0	0.73
Thyroid disease	17.4	20.0	0.39
Gastrointestinal diseases	17.8	17.5	0.91
Pre-diabetes	13.4	19.7	0.030
Hypertriglyceridemia	14.3	22.7	0.005
Eye disease	11.5	16.3	0.07
Physical impairment	11.6	10.8	0.75
Heart disease/stroke	8.1	4.7	0.06
Cancer	3.9	6.7	0.10
Fatty liver disease	7.6	11.5	0.09
Osteoporosis	4.6	13.6	<0.0001

© 2022 Mattei J et al. *JAMA Network Open.*

eTable 5. Age-Standardized Prevalence and Age-Standardized Means of Lifestyle and Psychosocial Risk Factors and Health Indicators of Adult Participants of the 2015 PRADLAD Study and Again in the Subsequent 2019 PROSPECT Study*

	**PRADLAD participants in 2015; *n* = 87**	**PRADLAD participants in 2019; n=87**	***P*-value**
	**Percent or mean (SD)**	**Percent or mean (SD)**	
Lifestyle factors			
Abdominal obesity	66.9	74.6	0.24
High waist-to-hip ratio	83.2	72.9	0.10
Self-rated poor/fair health	31.1	36.1	0.52
Self-rated poor/fair dietary habits	40.7	29.6	0.15
Sedentary physical activity	23.9	26.0	0.73
Current smoke	11.3	21.0	0.10
Current alcohol drinker	40.8	39.6	0.88
Binge drinker	19.6	23.2	0.58
Recurrent sleeping difficulties	19.7	19.0	>0.99
Extreme sleeping hours (<7h or >9h)	52.0	58.9	0.36
Yearly influenza vaccination	33.9	38.1	0.53
Psychosocial factors			
Depressive symptoms score	12.1 (1.7)	11.8 (1.5)	0.97
Depression-likely symptoms (≥16)	28.4	24.2	0.49
Perceived stress score	20.6 (1.0)	17.2 (1.2)	0.033
Social support score	27.6 (0.90)	27.8 (0.90)	>0.99
Appraisal	9.2 (0.36)	9.9 (0.32)	0.19
Belonging	9.5 (0.34)	8.8 (0.41)	0.14
Tangible	8.8 (0.33)	9.0 (0.37)	0.71
Self-reported medical diagnoses			
Hypertension	37.5	35.1	0.75
Anxiety	22.5	38.9	0.022
Obesity	33.6	43.3	0.16
Arthritis	23.7	22.2	0.72
Hypercholesterolemia	15.2	36.4	0.001
Depression	13.5	30.2	0.010
Respiratory problems	27.0	25.9	0.99
Diabetes	20.0	22.9	0.58
Thyroid disease	18.8	24.9	0.27
Gastrointestinal diseases	13.8	31.7	0.004
Pre-diabetes	20.2	16.4	0.43
Hypertriglyceridemia	14.6	17.6	0.68
Eye disease	13.5	19.5	0.31
Physical impairment	14.3	9.8	0.49
Heart disease/stroke	2.3	6.7	0.15
Cancer	3.8	7.5	0.19
Fatty liver disease	6.0	13.3	0.07
Osteoporosis	5.9	13.1	0.12

*Adjustment weights used to age-standardized estimates to the US 2010 population age distribution were as follows: <=39 years=0.396579, 40-59 years=0. 0.371795, ≥60 years=0.231626. Based on the 2010 US census population data obtained from: US Census Bureau. 2010 US Census summary file 1 singe years of age and sex. Available from: http://factfinder2.census.gov/faces/tableservices/jsf/pages/productview.xhtml?pid=DEC_10_SF1_QTP2&prodType=table (accessed June 2, 2021).

© 2022 Mattei J et al. *JAMA Network Open.*

eTable 6. Sociodemographic Characteristics of Adult Participants in the 2015 PRADLAD Study in Puerto Rico by Participation Status in the Subsequent PROSPECT Study[1]

**Characteristic**	**Not participating in PROSPECT (*n* = 293)***	**Participating in PROSPECT (*n* = 87)**	***P*-value**
	**Percent or mean (SD)**	**Percent or mean (SD)**	
Age, years	51.1 (11.4)	52.8 (10.5)	0.20
Sex-at-bith			
Female	63.8	71.3	0.20
Male	36.2	28.7	
Rural area of residence	17.2	11.5	0.20
Living alone	22.2	33.3	0.034
Puerto Rican ethnicity	80.9	83.9	0.64
Marital status			0.64
Married/living with partner	42.2	44.8	
Divorced/separated/widowed	22.0	17.3	
Single	35.8	37.9	
Educational attainment			0.23
Less than 8th grade	13.7	5.8	
9th–12th grade or GED	29.0	32.6	
Some college or college degree	47.4	48.8	
Graduate school	9.9	12.8	
Household income			0.27
$0–$10,000	60.4	58.4	
$10,001–$20,000	22.6	16.9	
>$20,001	17.0	24.7	
Employment			0.27
Currently employed	36.9	35.6	
Retired	46.4	54.0	
Unemployed	16.7	10.4	
Health insurance, %			0.56
Government-assisted	56.7	50.7	
Private	35.5	42.4	
Uninsured	7.8	6.9	
Food security and assistance			
Frequent food insufficiency	15.5	11.5	0.65
WIC food assistance	8.2	2.3	0.06
Government food assistance	54.4	40.2	0.021
Migration history, %			
Lived in PR most of their life	88.3	89.7	0.19
Lived in mainland US at least 1 year	30.7	18.4	0.026
Plans to move from PR	19.1	12.8	0.18

*Excludes 13 participants not age-eligible for the PROSPECT study and the 87 participants in both studies.

© 2022 Mattei J et al. *JAMA Network Open.*

eTable 7. Lifestyle and Psychosocial Risk Factors and Health Indicators of Adult Participants in the 2015 PRADLAD Study in Puerto Rico by Participation Status in the Subsequent PROSPECT Study

	**Not participating in PROSPECT (*n* = 293)***	**Participating in PROSPECT (*n* = 87)**	***P*-value**
	**Percent or mean (SD)**	**Percent or mean (SD)**	
Lifestyle factors			
Abdominal obesity	60.3	64.5	0.51
High waist-to-hip ratio	74.6	83.8	0.10
Self-rated poor/fair health	41.8	34.5	0.06
Self-rated poor/fair dietary habits	29.5	34.5	0.59
Sedentary physical activity	45.9	34.3	0.12
Current smoker	19.2	16.1	0.81
Current alcohol drinker	25.4	31.0	0.54
Binge drinker	11.3	14.9	0.36
Recurrent sleeping difficulties	21.6	23.3	0.10
Extreme sleeping hours (<7h or >9h)	49.1	50.0	0.88
Yearly influenza vaccination	22.9	34.9	0.08
Psychosocial factors			
Depressive symptoms score	18.7 (12.5)	14.2 (12.2)	0.006
Depression-likely symptoms (≥16)	57.5	36.4	0.001
Perceived stress score	22.2 (7.4)	20.4 (7.7)	0.06
Social support score	24.1 (7.2)	26.5 (6.8)	0.011
Appraisal	8.3 (2.8)	8.8 (2.7)	0.14
Belonging	7.9 (2.7)	9.1 (2.7)	0.0002
Tangible	7.8 (2.6)	8.7 (2.4)	0.010
Self-reported medical diagnoses			
Hypertension	39.0	40.20	0.87
Anxiety	30.0	28.6	0.80
Obesity	25.7	34.1	0.13
Arthritis	25.2	26.7	0.77
Hypercholesterolemia	25.3	19.1	0.24
Depression	23.1	18.38	0.35
Respiratory problems	20.1	23.5	0.49
Diabetes	20.0	23.3	0.51
Thyroid disease	17.4	18.8	0.76
Gastrointestinal diseases	17.8	15.1	0.57
Pre-diabetes	13.4	20.9	0.09
Hypertriglyceridemia	14.3	16.1	0.69
Eye disease	11.5	16.7	0.21
Physical impairment	11.6	16.5	0.24
Heart disease/stroke	8.1	2.3	0.06
Cancer	3.9	4.8	0.72
Fatty liver disease	7.6	7.4	0.96
Osteoporosis	4.6	7.0	0.39

*Excludes 13 participants not age-eligible for the PROSPECT study and the 84 participants in both studies.

© 2022 Mattei J et al. *JAMA Network Open.*

eTable 8. Sociodemographic Characteristics of Adult Participants of the PRADLAD Study in 2019 and of PROSPECT Participants in 2019 Recruited Through Other Strategies (Not PRADLAD)

	PRADLAD participants in 2019 (n=87)	Rest of PROSPECT participants (non-PRADLAD) in 2019 (n=447)	*P*-value
	Percent or mean (SD)	Percent or mean (SD)	
Age, years	56.4 (10.7)	53.2 (10.7)	0.012
Sex-at-birth			
Female	71.3	67.4	0.48
Male	28.7	32.6	
Rural area of residence	10.3	21.9	0.013
Puerto Rican ethnicity	83.9	93.8	0.002
Marital status			0.34
Married/living with partner	42.5	49.2	
Divorced/separated/widowed	32.2	24.9	
Single	25.3	25.8	
Educational attainment			0.31
Less than 8th grade	6.9	4.8	
9th–12th grade or GED	28.7	26.8	
Some college or college degree	55.2	51.8	
Graduate school	9.2	16.6	
Household income			0.15
$0–$10,000	41.8	34.8	
$10,001–$20,000	29.1	24.9	
> $20,001	29.1	40.3	
Employment			0.17
Currently employed	47.1	50.0	
Retired/stay-at-home	36.4	40.4	
Unemployed	16.5	9.6	
Health insurance			<0.0001
Government-assisted	49.4	34.2	
Private	44.8	60.6	
Uninsured	5.8	5.2	
Food security and assistance			
Frequent food insufficiency	13.4	11.8	0.68
WIC food assistance	4.3	5.9	0.56
Government food assistance	55.1	53.5	0.78
Migration history			
Lived in PR most of their life	93.1	95.3	0.39
Lived in mainland US at least 1 year	24.1	26.6	0.63
Plans to move from PR	3.5	5.9	0.37

© 2022 Mattei J et al. *JAMA Network Open.*

eFigure 1. Flowchart of 2015 PRADLAD Participants Who Were Recontacted and Eligible to Participate in the 2019 PROSPECT Study

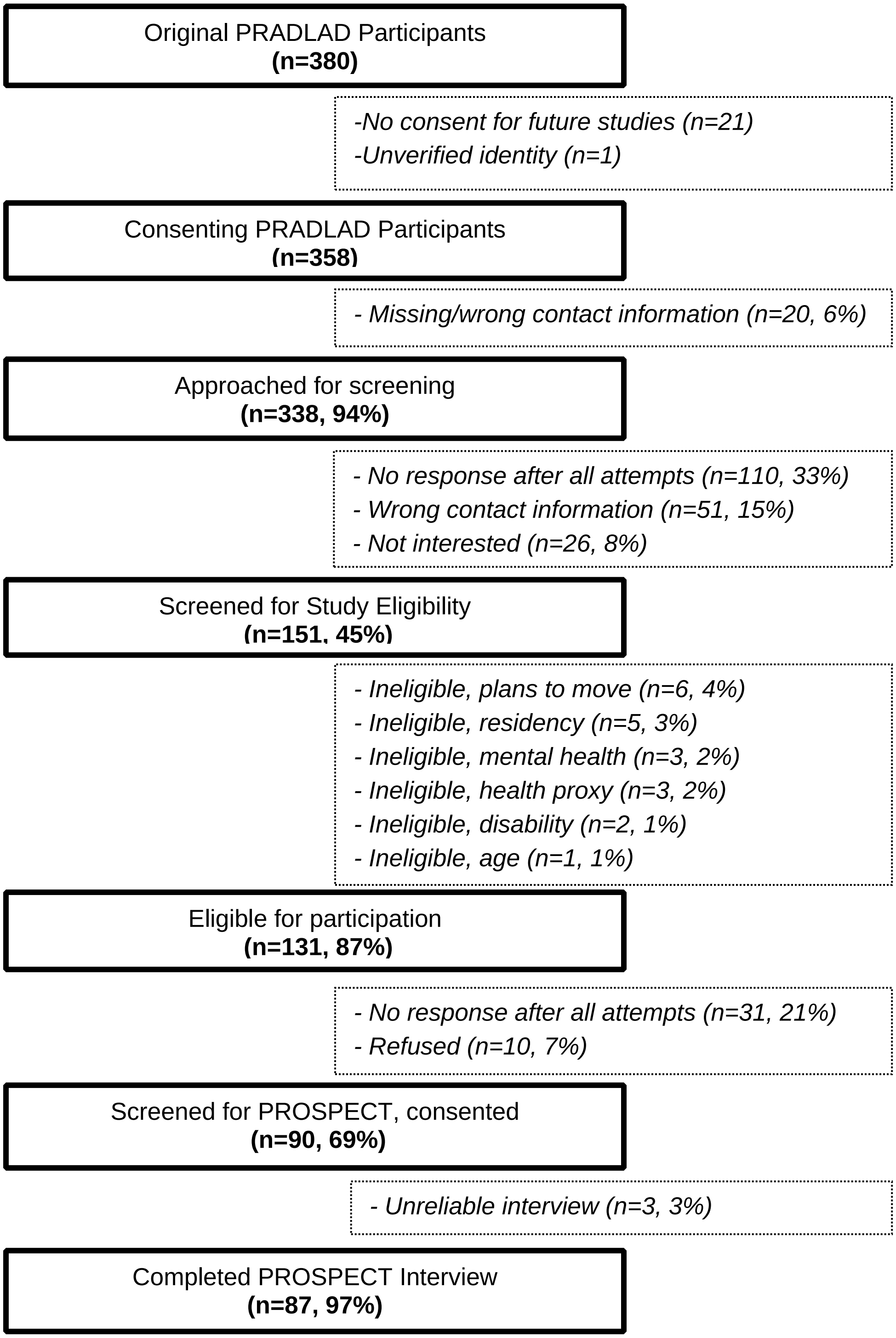

Legend: Unreliable interviews refer to participants deemed by a trained Research Assistant as providing confusing or inconsistent answers.

© 2022 Mattei J et al. *JAMA Network Open.*

eFigure 2. Flowchart of Recruitment of 2019 PROSPECT Participants as of March 16, 2020

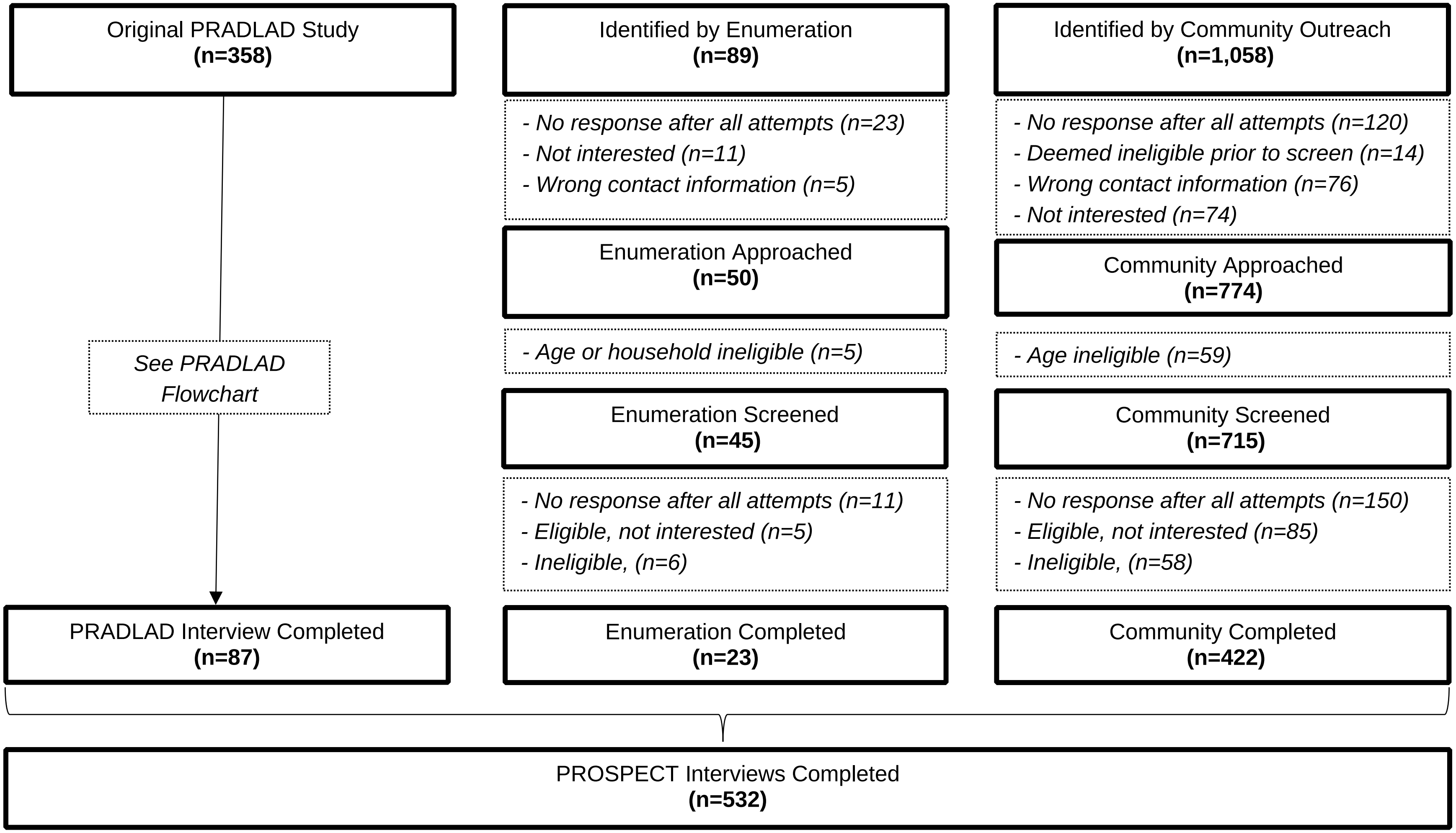

Legend: Enumeration was done by identifying households with potentially-eligible participants using 2010 Census block frames. While more households were identified, these were pending invitation to the study as of March 16th, 2020. Community outreach was done by advertising in partner clinics, community sites, media, and referrals.

© 2022 Mattei J et al. *JAMA Network Open.*
